# Weak interactions in crystals: an integrated approach

**DOI:** 10.1107/S2056989018004887

**Published:** 2018-04-17

**Authors:** Chiara Massera, Helen Stoeckli-Evans

**Affiliations:** a Dipartimento di Scienze Chimiche, della Vita e della Sostenibilità Ambientale, Università di Parma, Parco Area delle Scienze 17/A, 43124 Parma, Italy; b Institute of Physics, University of Neuchâtel, Rue Emile-Argand 11, CH-2000 Neuchâtel, Switzerland

**Keywords:** crystallography, weak interactions, editorial

## Abstract

Chiara Massera and Helen Stoeckli-Evans introduce this special issue of *Acta Crystallographica Section E* on ‘Weak interactions in crystals: an integrated approach’ where the contributions serve as an excellent introduction to the importance and the interest these have been acquiring in the scientific arena over the last two decades or so.

Since its appearance in 2001, and throughout subsequent years, *Acta Crystallographica Section E Crystallographic Communications* has been a benchmark for high-quality crystallographic data and structural discussions within the landscape of chemical scientific publications. The rigorous editing and reviewing of the papers, combined with the speed of publication and the open-access editorial policy makes the journal easily accessible to a very broad audience, encouraging the authors to place their results in a larger scientific context. The journal is now widening up its chemical content to encompass further different scientific fields related to crystallography, and to highlight hot and consolidated topics through application notes, themed issues and reviews.

The choice of weak interactions as topic for this special issue stems from the importance and the interest these have been acquiring in the scientific arena over the last two decades or so. Indeed, weak interactions have long been established as essential features in determining the chemical and physical properties of materials, influencing their three-dimensional structure, reactivity, organization, and biological activity. The number of papers and reviews dedicated to the crystallographic and theoretical study and analysis of hydrogen and halogen bonds, π–π interactions, and C—H⋯π interactions, just to cite a few, has grown exponentially over the years, underlining the applicability and usefulness of weak interactions to a wide range of scientific disciplines. Weak interactions are essential in the formation of inclusion compounds, in the self-assembly of host–guest molecular complexes, in the study of polymorphism, co-crystals, phase transitions and charge density. When crystallographic results are published it has become common practice to include an analysis of the crystal packing. As a matter of fact, the current format of *Acta E* papers encourages authors to include a section specifically dedicated to the supramolecular features of the molecules studied.

In the present special issue, we have aimed to collect a series of examples showing the fundamental role of detailed structural analysis in understanding weak interactions in the solid state, introduced by a general review by Batsanov, which highlights the history, state-of-the art and future developments of this field. The articles comprise six *Research Communications* on molecular salts (Blignaut & Lemmerer; Canossa *et al.*), organic compounds (Geiger *et al.*, Dey *et al.*), as well as host-guest (Yamada *et al.*) and coord­ination complexes (Seth), in which the weak interactions reported have in many cases been analysed by a combination of crystallographic analysis and theoretical techniques, showing the importance of an integrated approach. The role of different experimental set ups when collecting data has been highlighted in an *Application Note* (Zakharov *et al.*), the first of a new category of articles for *Acta E* that will highlight the benefits of choosing a particular instrument or piece of equipment or using a particular data-collection strategy.

We would like to thank all the authors who, with their contributions, have made this special issue possible, as well as the editorial staff in Chester for their constant help and advice.

